# Flame-Made Calcium Phosphate Nanoparticles with High Drug Loading for Delivery of Biologics

**DOI:** 10.3390/molecules25071747

**Published:** 2020-04-10

**Authors:** Vasiliki Tsikourkitoudi, Jens Karlsson, Padryk Merkl, Edmund Loh, Birgitta Henriques-Normark, Georgios A. Sotiriou

**Affiliations:** 1 Department of Microbiology, Tumor and Cell Biology, Karolinska Institutet, SE-1 71 77 Stockholm, Sweden; vasiliki.tsikourkitoudi@ki.se (V.T.); jens.karlsson@ki.se (J.K.); padryk.merkl@ki.se (P.M.); edmund.loh@ki.se (E.L.); birgitta.henriques@ki.se (B.H.-N.); 2 Lee Kong Chian School of Medicine (LKC) and Singapore Centre on Environmental Life Sciences Engineering (SCELSE), Nanyang Technological University, Singapore 639798, Singapore; 3 Department of Clinical Microbiology, Karolinska University Hospital, SE-171 76 Stockholm, Sweden

**Keywords:** nanocarriers, drug delivery, biologics, calcium phosphate, flame spray pyrolysis, LL-37, stability, antimicrobial properties

## Abstract

Nanoparticles exhibit potential as drug carriers in biomedicine due to their high surface-to-volume ratio that allows for facile drug loading. Nanosized drug delivery systems have been proposed for the delivery of biologics facilitating their transport across epithelial layers and maintaining their stability against proteolytic degradation. Here, we capitalize on a nanomanufacturing process famous for its scalability and reproducibility, flame spray pyrolysis, and produce calcium phosphate (CaP) nanoparticles with tailored properties. The as-prepared nanoparticles are loaded with bovine serum albumin (model protein) and bradykinin (model peptide) by physisorption and the physicochemical parameters influencing their loading capacity are investigated. Furthermore, we implement the developed protocol by formulating CaP nanoparticles loaded with the LL-37 antimicrobial peptide, which is a biological drug currently involved in clinical trials. High loading values along with high reproducibility are achieved. Moreover, it is shown that CaP nanoparticles protect LL-37 from proteolysis in vitro. We also demonstrate that LL-37 retains its antimicrobial activity against *Escherichia coli* and *Streptococcus pneumoniae* when loaded on nanoparticles in vitro. Therefore, we highlight the potential of nanocarriers for optimization of the therapeutic profile of existing and emerging biological drugs.

## 1. Introduction

Biological drugs, also called biologics, are therapeutics that contain one or more active substances made by or derived from a biological source [1]. Biologics include proteins, antibodies, peptides, nucleotides and are an attractive class of therapeutic moieties for long-term medical illnesses/conditions with no other treatment available. Approximately 31% of all drugs approved by the US Food and Drug Administration (FDA) during the last 5 years were biologics [2]. Biologics exhibit high potency coupled with target specificity, however, there are some important challenges for their broad and effective employment: (i) their transport across various epithelial layers, such as skin and mucus, is limited due to their large size, (ii) they typically suffer from poor bioavailability via the oral route and, thus, can only be administered systemically (e.g., by injection) [3], (iii) they are susceptible to enzymatic degradation in tissues and plasma and consequently their circulation half-life is shortened.

To address these challenges, nanosized drug delivery systems have been proposed as a promising strategy for the delivery of biologics [4]. Nanoparticles can be used as drug carriers due to their high surface-to-volume ratio (large surface area/small size) that allows for their interaction with other similarly-sized biological entities. Most common nanocarriers in the literature are based on organic materials (e.g., liposomes, dendrimers, lipid nanoparticles) [5]. However, the mechanism of degradation of these organic nanomaterials in biological systems is not fully understood raising concerns regarding the biocompatibility of their degradation products [6].

Recently, the development of novel inorganic nanocarriers has gained significant attention and is considered among the fastest growing areas of drug delivery [7,8]. Phosphate-based nanocarriers have shown great potential due to their solubility in aqueous or slightly acidic conditions, their biodegradability and biocompatibility. They consist of divalent cations (usually Ca^2+^ [9], Mg^2+^ [10], Mn^2+^ [10,11], Zn^2+^ [12]) that form ionic complexes with macromolecules, that in turn can be easily transferred across cell membranes via ion channel mediated endocytosis [13]. Among them, calcium phosphate (CaP) nanoparticles are an important family of biomaterials in drug delivery because of their excellent biocompatibility, low toxicity, non-immunogenicity and osteoconductivity. In fact, the high biocompatibility of CaP nanoparticles renders it advantageous compared to polymeric, lipid-based, or other metal-based nanocarriers that may cause adverse effects and are not always biodegradable, and thus pose a risk of accumulation in the body [14]. The high biocompatibility of CaP nanoparticles is further supported by the fact that CaP is approved by the US FDA and is clinically available as bone graft formulation (Gem21S^®^, Osteohealth). In this formulation, CaP acts both as a carrier of a biological drug (platelet-derived growth factor) and as a bone tissue regeneration matrix. This has prompted studies with CaP as a potential carrier for several biologics such as proteins [15], peptides [16,17,18] and nucleotides [9,19,20] against various diseases.

Despite the current scientific interest of nanoparticle-based therapeutics, there are still barriers for their clinical translation with the most important ones summarized below [21]:

(1) Complex synthesis methods of nanocarriers, that require multiple individual preparation steps, cannot be easily scaled up and reproduced leading to batch-to-batch inconsistencies.

(2) Poor loading efficiency of biological drugs in comparison to clinically relevant therapeutic doses. Many methods do not permit facile tuning of nanocarrier properties, such as specific surface area (SSA) and size, that directly affect drug loading. Typical loading values for liposomes are in the range of 1%–10%, while for polymeric nanocarriers loading values do not surpass 20% [22,23]. Loading efficiency is a crucial factor determining the excipient amount in nanopharmaceutical products (i.e., nanoparticles in this case). In case of low loading, a larger amount of nanoparticles is needed in order to deliver a clinically relevant dose of the drug. However, an increase of the excipient amount might cause undesirable side effects and increase the manufacturing cost [5].

(3) Stability of the biological drug due to possible loss of its biological functionality during the loading process.

Technological developments for overcoming existing barriers are important to enable the future success of nanoformulations for drug delivery.

Here, we aim to address these challenges and engineer inorganic drug delivery nanocarriers by flame spray pyrolysis (FSP). We exploit the versatility of this scalable and reproducible nanomanufacturing process for the synthesis of CaP nanocarriers with tailored properties. FSP can produce multi-component nanoparticles with high control over their specific surface area (SSA)/size, composition and crystallinity in a single step [24,25]. A distinct target here is to maximize the biological drug loading on these nanocarriers. There are still large variabilities in the drug loading capacity of CaP nanocarriers described in the literature [26], highlighting the need for an improved protocol for the loading of biological drugs on the surface of nanocarriers. We develop here such an experimental protocol for loading biological drugs (proteins and peptides) on flame-made CaP nanoparticles by physisorption, an increasingly popular route for nanoparticle biofunctionalization [27]. We study the loading on CaP nanoparticles of two different SSA/sizes of two biomacromolecules, bovine serum albumin (BSA) and bradykinin as model protein and peptide, respectively. We therefore address challenges regarding insufficient stability, low drug loading capacity, expensive manufacturing and poor yield and unclear biocompatibility [28].

In order to further implement the developed protocol in biological drugs currently in use, we utilized the LL-37 antimicrobial peptide, currently being explored in several clinical trials for its antimicrobial, wound healing and immunomodulatory properties (see ClinicalTrials.gov Identifier: NCT02225366 & NCT04098562) [29]. LL-37 has a net positive charge at physiological conditions, is amphiphilic and can eliminate the pathogenic microbes directly via electrostatic attraction towards negatively charged bacterial membranes [30]. Upon its immobilization on nanoparticle surfaces, LL-37 exhibits bactericidal activity against various bacteria [31,32,33,34]. Braun et al. [31] showed the effect of surface charge and SSA of mesoporous SiO_2_ nanocarriers on loading and release of LL-37 and they investigated their antimicrobial activity against *Escherichia coli* 25922. M. Vignoni et al. [35] investigated the antimicrobial behaviour of LL-37-loaded Ag nanoparticles in skin infections and its antibiofilm formation activity. In this case, they did not observe any bactericidal effect of LL-37 for *Pseudomonas aeruginosa*, *Staphylococcus epidermidis* and *Staphylococcus aureus*. The antibacterial effect was attributed to Ag nanoparticles, whereas LL-37 promoted proliferation of skin fibroblasts. Garcia-Orue et al. [36] demonstrated the antimicrobial activity of LL-37 encapsulated in nanostructured lipid carriers (NLC) against *E. coli*. These nanocarriers had ~73% bacterial killing and significantly improved wound healing in vivo (mice) in comparison to free LL-37. Cherredy et al. [37] also investigated wound healing properties of LL-37 encapsulated in polymeric nanoparticles, and more precisely in poly (lactic-co-glycolic acid) (PLGA). Although PLGA-LL-37 formulation was more efficient in wound healing than free LL-37, it did not have any significant antimicrobial activity against *E. coli* as even at the highest LL-37 concentration tested (5 μg/mL), the survival of bacterial cells was ~70%. To the best of our knowledge, there are no studies so far demonstrating the use of CaP nanoparticles as LL-37 carriers. Exploring the current trend of developing novel antibiotics by antimicrobial peptides, we formulate LL-37-loaded CaP nanoparticles with high loading and high reproducibility. We further study the stability of the peptide upon its loading on CaP nanoparticles against proteinase K and investigate the antimicrobial activity against *E. coli* and *Streptococcus pneumoniae*.

## 2. Results and Discussion

### 2.1. Particle Morphology

Flame aerosol nanoparticle synthesis allows for a fine control over the product nanoparticle primary size by tuning the process conditions. More specifically, by controlling the precursor concentration and the flow rate of the dispersion gas (O_2_ in this case) during FSP, CaP nanoparticles of different SSA are obtained, as measured by N_2_ adsorption. Table 1 shows the process conditions for the two samples made here and the resulting SSA. The average primary particle diameter, d_BET_, is calculated from the SSA assuming monodisperse solid spherical particles with density of 3.16 g/cm^3^ [38]. Increasing precursor concentration during FSP yields larger primary particle sizes attributed to the longer particle residence time at high temperatures [39].

**Figure 1:**
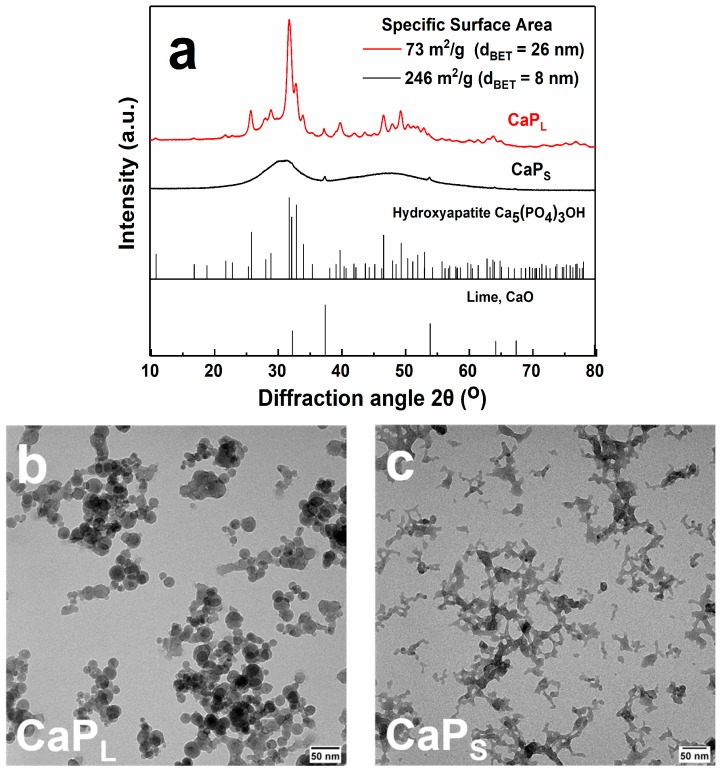
(**a**) X-ray diffraction (XRD) patterns of the CaP nanoparticles. The specific surface area (SSA), as determined by the nitrogen adsorption–desorption isotherms, along with the corresponding primary particle size, d_BET_, are also shown. By varying FSP synthesis conditions either crystalline or amorphous particles are obtained. Main peaks are assigned to hydroxyapatite, Ca_5_(PO_4_)_3_OH, whereas CaO is also observed. Transmission electron microscopy (TEM) images of as-prepared CaP_L_ (**b**) and CaP_S_ (**c**) samples. CaP_L_ particles are spherical with loosely agglomerated structure, while fused particles with sintered necks are clearly illustrated for CaP_S_ (Scale bar 50 nm).

The crystallinity of the as-prepared nanoparticles is evaluated by X-ray powder diffraction (XRD) and shown in Figure 1a, in which the characteristic hydroxyapatite (ICSD: 151414) and CaO (ICSD: 26959) crystal phases are also present. Broad reflections are shown for the CaP_S_ sample, indicating the amorphous nature of the particles while sharp peaks are exhibited for the CaP_L_ sample. Further minor sharp peaks corresponding to CaO exist in both samples (<4wt% as determined by Rietveld analysis). Therefore, by controlling the precursor concentration within the flame, the crystallinity of the product nanoparticles is finely tuned from completely amorphous to highly crystalline, in agreement with the literature [40,41]. Representative transmission electron microscopy (TEM) images of both CaP_L_ and CaP_S_ nanoparticles are shown in Figure 1b and c, respectively. The characteristic fractal-like structure of flame-made nanoparticles consisting of agglomerates and/or aggregates with several primary particles within each agglomerate/aggregate is shown.

### 2.2. Loading Macromolecules on CaP Nanoparticles: Effect of Incubation Time and Macromolecule Concentration

**Figure 2:**
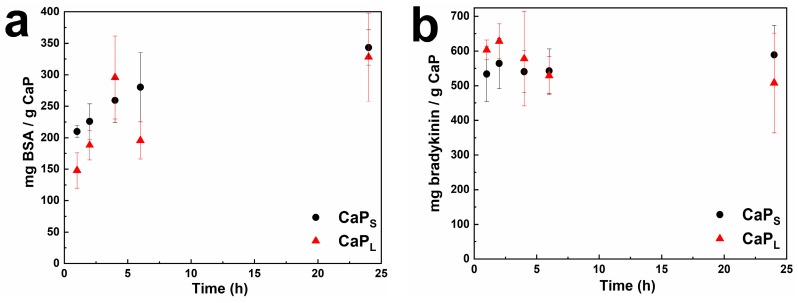
Effect of incubation time on the loading capacity of CaP_S_ and CaP_L_ nanoparticles for (**a**) bovine serum albumin (BSA); and (**b**) bradykinin (pH 7.4, particle concentration 500 μg/mL, BSA/bradykinin concentration 500 μg/mL). Both BSA and bradykinin rapidly adsorb on the nanoparticles’ surface during the first hour.

The loading capacity of macromolecules on flame-made CaP nanoparticles by physisorption is studied with BSA and bradykinin as a model protein and peptide, respectively. BSA and bradykinin are incubated in phosphate-buffered saline (PBS) (pH 7.4) at room temperature along with both CaP samples for up to 24 h and constant particle and protein concentration (both at 500 μg/mL). The effect of incubation time on the loading capacity is shown in Figure 2a,b, respectively. BSA and bradykinin rapidly physisorb on the nanoparticle surface during the first hour. The BSA loading capacity of both CaP_S_ and CaP_L_ nanoparticles increases over time. CaP_S_ particles with higher SSA demonstrate slightly higher values for the same mass concentration than CaP_L_ that are larger and have lower SSA (Table 1). The difference in loading capacity for the two samples might be attributed to the accessible surface area and thus the availability of binding sites between BSA and CaP nanoparticles; however, it is not proportional to the nominal SSA values of the two samples. This may be attributed to both the agglomeration/flocculation that occurs upon dispersion in liquids, but also because of the differences in crystallinity, as charged molecules might adsorb differently on surfaces depending on their crystal phase [42]. Agglomeration is energetically favorable and it is challenging to avoid in colloidal systems without any surface treatment (e.g., surface functionalization or coating) [43]. Bradykinin loading for both CaP_s_ and CaP_L_ reaches steady-state earlier than BSA probably due to its smaller size and the loading values do not differ significantly over the 24 h studied here. Nonetheless, the loading values for BSA and bradykinin reach 350 mg/g and 600 mg/g of CaP that further highlights the high loading capacity of the flame-made CaP nanoparticles. Because of the slightly higher loading capacity values exhibited from the CaP_S_ sample, further results here focus on this size.

**Figure 3:**
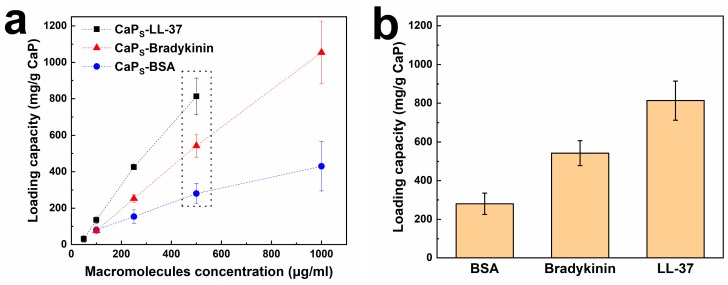
(**a**) Effect of concentration of BSA, bradykinin and LL-37 on the loading capacity of CaPs nanoparticles (pH 7.4, particle concentration 500 μg/mL); (**b**) Loading capacity of CaPs nanoparticles for particle concentration 500 μg/mL and macromolecule concentration (i.e., BSA, bradykinin and LL-37) 500 μg/mL. Data are reported as mean ± standard deviation, for at least 3 independent triplicates.

Figure 3a shows the loading capacity of CaP_S_ nanoparticles for BSA (blue circles) and bradykinin (red triangles) after incubation for 6 h as a function of the initial macromolecule concentration (pH 7.4 and particle concentration of 500 μg/mL, please see Supplementary Materials, Figure S1 for CaP_L_). By increasing BSA and bradykinin concentration in the range of 100 to 1000 μg/mL, the adsorbed quantity on the nanoparticles monotonically increases. The BSA/bradykinin concentration has not reached saturation conditions up to 1000 μg/mL indicating that this concentration range is still in the linear part of the Langmuir isotherms [44]. Furthermore, the same procedure is employed to load LL-37 antimicrobial peptide on CaP_S_ nanoparticles (black squares in Figure 3a, incubation for 4 h in PBS). Similar to BSA and bradykinin, the loaded amount increases for increasing LL-37 concentration. LL-37 exhibits more efficient physisorption than BSA and bradykinin at the same initial concentrations as shown in Figure 3b. This is probably attributed to the more cationic charge of LL-37 than BSA/bradykinin that enables LL-37 to readily physisorb on the CaP nanoparticle surface. The maximum LL-37 loading achieved here reaches ~800 mg/g CaP, that outperforms all LL-37 loadings found in the literature (Table 2). This high loading is probably attributed to the fractal-like agglomerated/aggregated structure that is typical for flame-made nanomaterials (Figure 1b,c). We also show in Table 2 the minimum inhibitory concentration (MIC) values of LL-37 and of the nanoparticle dose (in μg/mL), as will be discussed later on.

**Figure 4:**
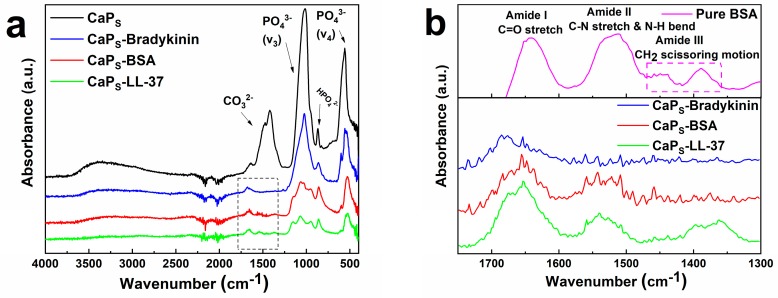
(**a**) Fourier-transform infrared spectroscopy (FTIR) spectra of CaP_S_ and freeze-dried bradykinin, BSA and LL-37-loaded CaP_S_ nanoparticles showing the characteristic absorption bands for phosphate chemical groups; (**b**) Magnified amide bond region indicating the presence of new peaks in the spectra of loaded samples, that represent amide I, amide II and amide III bonds.

In order to further study the presence of the macromolecules on the CaP_S_ nanoparticle surface, Fourier-transform infrared spectroscopy (FTIR) analysis is performed on freeze-dried nanoparticles loaded with the biologics (Figure 4a). The successful physisorption of macromolecules onto the nanoparticle surface is confirmed in Figure 4b, that shows a magnification of the spectra, by the presence of new peaks around 1650–1680 cm^−1^, 1540 cm^−1^ and 1360 cm^−1^, representing amide I, amide II and amide III bonds, respectively, in agreement with the literature. More precisely, amide I peaks are mainly associated with C=O stretching vibrations, whereas amide II peaks are correlated to C–N stretching and N–H bending. Amide III bonds result from CH_2_ scissoring motion [44]. Moreover, as shown in Figure 4a, when the spectra of loaded particles (red, blue and green lines) are compared to the spectrum of pure CaP_S_ nanoparticles (black line), there is a decrease of the intensity of the PO_4_^3−^ bands and the disappearance of CO_3_^2−^ band. Even though the CO_3_^2−^ from the amorphous CaP_S_ might dissolve in aqueous solutions resulting in the disappearance of that band, the reduction of the PO_4_^3−^ peak along with slight shifting of the peaks of pure BSA to higher wavenumbers for the loaded CaP_S_ (green, red and blue lines in comparison to magenta line in Figure 4b) indicate that there is some interaction at these sites between the CaP and the macromolecules [47,48].

**Figure 5:**
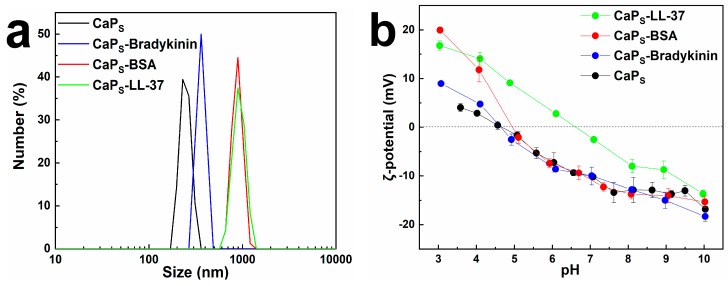
(**a**) Size distribution of CaP_S_ nanoparticles (number % data) before and after loading with BSA, bradykinin and LL-37 in phosphate-buffered saline (PBS) pH 7.4, as determined by dynamic light scattering (DLS) measurements (particle concentration 100 μg/mL); (**b**) *ζ*-potential profile of CaP_S_ and loaded CaP_S_ particles as determined by titration at different pH (particle concentration 100 μg/mL).

The hydrodynamic size measured by dynamic light scattering (DLS) in PBS (particle concentration 100 μg/mL) before and after macromolecule loading on the CaP_S_ nanoparticles is shown in Figure 5a (number distribution, please see Figure S2 in Supplementary Materials the intensity distribution). The presence of protein/peptide seems to promote nanoparticle agglomeration due to increased agglomerate–agglomerate interactions. The developed nanocarriers here form agglomerates when suspended in solution in agreement with similar flame-made nanoparticles [49] and their sedimentation occurs over a few hours (Supplementary Material, Figure S5). Even though small hydrodynamic sizes are typically preferred for intravenous delivery, larger sizes might also be suitable for other administration routes, such as pulmonary (e.g., dry powder inhaler) or local administration (e.g., wound healing). Furthermore, the effect of pH on ζ-potential values of the CaP_S_ nanoparticles is shown in Figure 5b (size distribution and *ζ*-potential profile of CaP_L_ are shown for comparison in Supplementary Materials, Figure S3, whereas the effect of pH on *ζ*-potential values of pure BSA, pure bradykinin and pure LL-37 are shown in Supplementary Materials, Figure S4). The isoelectric point (IEP) of pure CaP_S_ is determined to be ~4.7, whereas when loaded with BSA and bradykinin there is practically no change probably because of the similar IEP of pure BSA and bradykinin, respectively (Supporting Information, Figure S4). However, there is a significant increase of the IEP of CaP_S_ particles to ~7, when they are loaded with LL-37, indicating the presence of LL-37 on the CaP_S_ surface.

### 2.3. Assessement of LL-37 Stability and Antimicrobial Assays

#### 2.3.1. LL-37 Loading on CaP Nanoparticles Keeps LL-37 Intact from Degradation by Proteinase K

**Figure 6:**
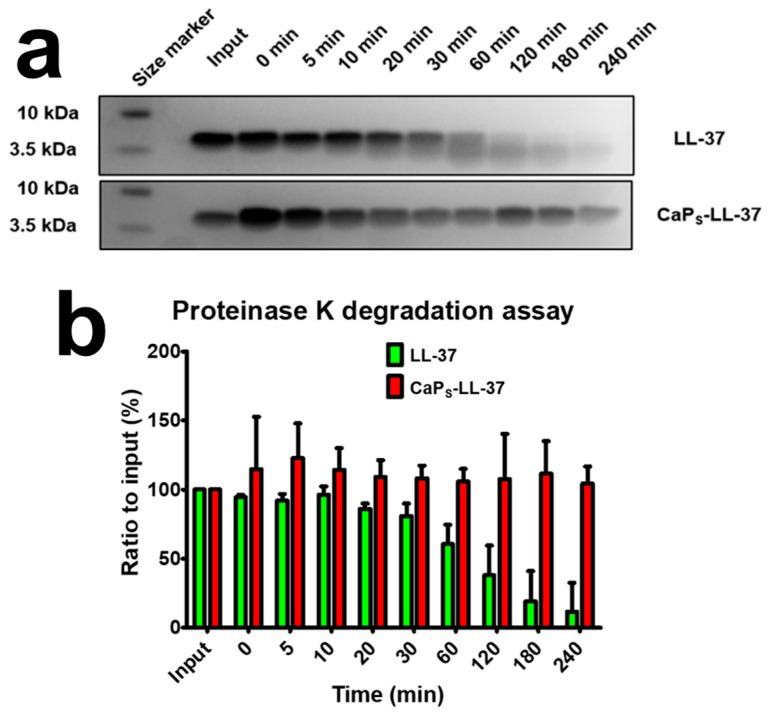
Proteinase K degradation assay of LL-37 in 20 mM Tris-HCl, pH 8.0 with a total of 20 ng Proteinase K. (**a**) Representative Sodium Dodecyl Sulfate–Polyacrylamide Gel Electrophoresis (SDS-PAGE) gels before and after proteolytic degradation of pure LL-37 and LL-37-loaded on CaP_S_ nanoparticles. A clear LL-37 band can be seen at a size of 4.5 kDa which starts degrading steadily after 20 min incubation, however, when loaded on CaP_S_, the LL-37 resists degradation up until 240 min incubation. Input is the pure LL-37 or LL-37-loaded on CaP_S_ nanoparticles (CaP_S_-LL-37) without any proteinase K added. Gel pictures shown are representative of at least three repetitions; (**b**) Quantification of bands for numerical assessment of degradation. Error bars are shown as standard deviation (N = 3).

Maintaining stability against enzymatic degradation and retaining biological functions of macromolecular drugs is crucial for biologics delivery systems. In order to assess the enzymatic degradation of LL-37 and the protective potential from the CaP_S_ nanocarriers, a degradation assay utilizing Proteinase K was performed (Sodium Dodecyl Sulfate–Polyacrylamide Gel Electrophoresis, SDS-PAGE). Figure 6a shows a representative gel electrophoresis of the pure LL-37 and the LL-37 loaded on the CaP_S_ nanoparticles after incubation with Proteinase K for up to 240 min. A clear LL-37 band can be seen at a size of 4.5 kDa which starts fading steadily after 20 min incubation in the case of pure LL-37 and new smears appear at lower molecular weight. The fading of the LL-37 band is attributed to its degradation over time because of proteolysis by proteinase K. LL-37 degradation by proteinase K has also been shown by Oren et al. (uncleaved peptide after 2 h ~ 5%) [50]. However, when loaded on CaP_S_, the LL-37 fully resists degradation up until 240 min incubation. Figure 6b shows the quantification of these bands demonstrating that loading on CaP_S_ offers protection of LL-37 against proteolytic degradation. This is in line with Braun et al. [31] who showed protection of LL-37 by *P. aeruginosa* elastase and leucocyte elastase when loaded on mesoporous SiO_2_ nanoparticles, and further highlights that such a macromolecule protection from enzymatic degradation occurs not only from mesoporous materials but also from fractal-like agglomerates/aggregates.

#### 2.3.2. Antimicrobial Activity of LL-37-Loaded CaP_S_

**Figure 7:**
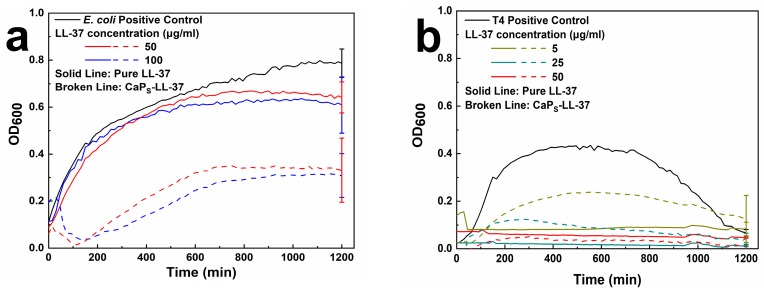
Growth curves (OD_600_ vs. time) of (**a**) *E. coli* (HVM52); (**b**) *S. pneumoniae* (T4), after subtraction of background (Figure S6). Measurements had been performed at least in triplicate and mean values are presented with representative error bars.

To verify if the LL-37 retains its antimicrobial function after being physisorbed onto CaP_S_ nanoparticles, its antimicrobial activity was studied against two bacterial pathogens, *E. coli* and *S. pneumoniae*. Figure 7 shows the background-subtracted (see Supplementary Materials for background signals, Figure S6) optical density at λ = 600 nm (OD_600_) as a function of time for 20 h of cultures containing the *E. coli* (a) and the *S. pneumoniae* serotype 4 strain TIGR4 (T4) (b) incubated either with the pure LL-37 or the LL-37-loaded nanoparticles for concentrations of LL-37 = 50–100 μg/mL for *E. coli* and 5–50 μg/mL for *S. pneumoniae*. The OD_600_ values directly correlate to the concentration/population of the bacterial cultures [51]. Figure 7a shows that pure LL-37 barely inhibits the *E. coli* growth up to 100 μg/mL, but most importantly that the LL-37-loaded nanoparticles also exhibit antimicrobial activity with the ones of the highest LL-37 concentration exhibiting better performance than the pure peptide. This might be attributed to the protective effect of CaP_S_ nanoparticles to LL-37 from secreted proteinases by the pathogens during the in vitro assay, leading to higher active concentration of LL-37 when it is loaded on the CaP_S_ nanoparticles than when it is free. The antimicrobial effect is even more pronounced against *S. pneumoniae* (Figure 7b), in which both pure LL-37 peptide and the LL-37-loaded nanoparticles completely inhibit the bacterial growth at LL-37 concentration of 50 μg/mL. At LL-37 concentration of 25 μg/mL, CaP_S_-LL-37 induce early lysis of the bacterial cell. At the lowest concentration (5 μg/mL), pure peptide seems to be more effective than peptide-loaded nanoparticles, as it completely inhibits bacterial growth, whereas CaP_S_-LL-37 inhibit bacterial growth up to 50% (as maximum OD_600_ attained, ~0.2, is approximately half of the maximum OD_600_ attained for the positive control, ~0.4). The growth curve of T4 (positive control, black line in Figure 7b) is typical for *S. pneumoniae*, as the OD_600_ decrease after reaching maximum values is attributed to autolysis of the pathogen [52].

**Figure 8:**
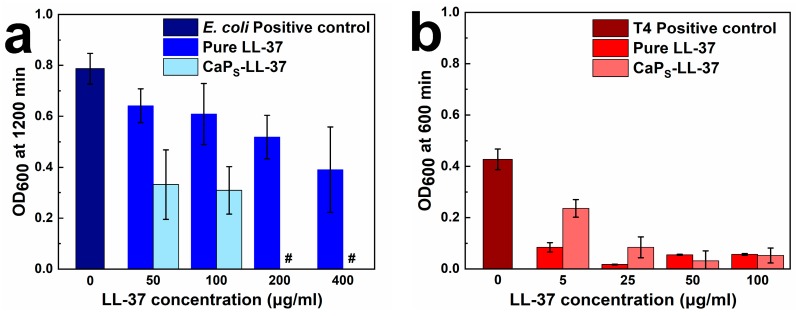
OD_600_ values at (**a**) 1200 min for *E. coli* (HVM52); (**b**) 600 min for *S. pneumoniae* (T4) for all LL-37 concentrations tested. Measurements had been performed at least in triplicate. Mean values after subtraction of background (Figure S6) are presented. In (**a**), # denotes negative OD_600_ due to background subtraction (plotted as zero in the graph for simplicity).

In Figure 8, the OD_600_ values for all LL-37 concentrations tested are presented for *E. coli* (a) and *S. pneumoniae* (b). For *E. coli* (Figure 8a), the antimicrobial activity of LL-37-loaded CaP_S_ nanoparticles outperforms the activity of pure peptide for all the concentrations tested. OD_600_ values for LL-37 concentrations of 200 and 400 μg/mL for CaP_S_-LL-37 conjugates are shown as zero as after background subtraction they are negative. For *S. pneumoniae* (Figure 8b), the antimicrobial activity of CaP_S_-peptide conjugates is similar to that of the pure peptide at concentrations higher than 5 μg/mL. The presence of pure particles did not exhibit any growth inhibition and the observed antibacterial activity is attributed to the LL-37 peptide and not to the CaP_S_ nanoparticles (see Supplementary Materials, Figure S7). The fact that LL-37 and LL-37-loaded CaP_S_ are more effective towards *S. pneumoniae* than *E. coli* was expected as *E. coli* (and other Gram-negative bacteria, like *P. aeruginosa*) is more resistant towards LL-37 than *S. pneumoniae* (Gram-positive) [53,54]. Most importantly, these results highlight that the loading of LL-37 onto the flame-made nanoparticles fully retains the peptide functionality and highlights the potential of these nanoparticles as biological drug nanocarriers. Furthermore, from these graphs the MIC values of the LL-37-loaded CaP_S_ may also be calculated as shown in Table 2. Upon comparing the MIC values from the present study and those from literature in Table 2, it is highlighted that low LL-37 MIC values do not necessarily mean a low nanocarrier dose, and further showing that the high LL-37 loading values achieved here facilitate the clinical translation of flame-made CaP nanoparticles as biological drug nanocarriers.

**Figure 9:**
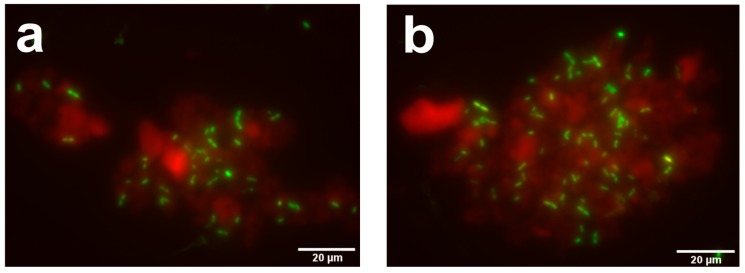
Representative z-stack projection images acquired by fluorescence microscopy showing *E. coli* (in green) (LL-37 concentration 400 μg/mL) attached on LL-37-loaded CaP_S_ agglomerates (in red) after 2 h incubation time at 37 °C.

The LL-37 release from the CaP_S_-LL-37 nanoconjugates at 25 °C and 37 °C, at pH 7.4 after 2 h, 6 h and 24 h is further studied in which we observe negligible release, <2% for both temperatures (Table S2 in the Supplementary Materials) indicating that the antibacterial activity is mainly attributed to direct bacterial contact with the nanoparticles. In order to further investigate the antibacterial mechanism of the CaP_S_-LL-37 nanoparticles, we acquire images by fluorescence microscopy of bacterial cultures tracking both the bacteria (stained green by SYTO9 Green Fluorescent Nucleic Acid Stain that labels both live and dead bacteria) and the nanocarriers (stained red by their intrinsic luminescence due to the Eu^3+^ ion doping, please see Materials and Methods, Section 3.1) after incubation for 2 h at 37 °C with the LL-37-loaded CaP_S_ nanoparticles (LL-37 concentration 400 μg/mL). The images in Figure 9 show *E. coli* (green) attached onto the CaP_S_-LL-37 agglomerates (red). We could not detect any *S. pneumoniae* T4 bacteria at these conditions indicating that all bacteria have been lysed already (images not shown). This further indicates that the antibacterial mechanism is mainly through direct bacterial contact with the nanoparticles.

## 3. Materials and Methods

### 3.1. Particle Synthesis and Characterization

CaP nanoparticles doped with 5 at% europium (vs. Ca) were produced by FSP. The Eu-doping enabled the detection of the CaP nanoparticles by monitoring their luminescence. Initially, calcium and europium precursors, i.e., calcium acetate hydrate (≥ 99%, Sigma-Aldrich, Stockholm, Sweden) and europium nitrate hexahydrate (99.9%, Alfa Aesar, Kandel, Germany), respectively, were added in the solvent mixture comprised by 2-ethylhexanoic acid (99%, Sigma-Aldrich, Stockholm, Sweden) and propionic acid (≥ 99.5%, Sigma-Aldrich, Stockholm, Sweden) in 1:1 ratio and stirred under reflux for 30 min at 70 °C. Subsequently, tributyl phosphate (≥ 99%, Sigma-Aldrich, Stockholm, Sweden) was added (phosphorus precursor), after clear solution was observed, in appropriate quantity in order to obtain Ca/P molar ratio of 2.19. A high Ca/P molar ratio is chosen in order to prevent dissolution of the particles, which highly depends on Ca/P molar ratio (increased dissolution is observed with decrease of Ca/P molar ratio). The total metal concentration of the precursor solution was 0.1 M or 0.2 M. The precursor solution was delivered to the flame through a capillary tube (SGE Analytical Science, Milton Keynes, UK) using a syringe pump (New Era Pump Systems, Inc., Farmingdale, NY, USA). The solution was atomized in the FSP nozzle by oxygen gas (> 99.5%, Linde AGA Gas AB, Stockholm, Sweden) (EL-FLOW Select, Bronkhorst, Ruurlo, Netherlands) at constant pressure (1.8 bar). The synthesis of the particles was carried out at X:Y 3:8 and 8:3 where X is the ratio of the precursor feed flow rate (mL/min) and Y is the O_2_ dispersion gas flow rate (L/min). The spray flame was ignited by a premixed supporting flame of methane/oxygen (> 99.5%, Linde AGA Gas AB, Stockholm, Sweden) at flow rates of 1.5 L/min and 3.2 L/min, respectively. The particles were collected on a glass fiber filter (Albet LabScience, Dassel, Germany) with the aid of a Mink MM 1144 BV vacuum pump (Busch, Mölnlycke, Sweden).

Phase identification of the as-prepared powders was performed by X-Ray diffraction (XRD). XRD data were collected at ambient temperature with a MiniFlex X-ray diffractometer (Rigaku Europe, Neu-Isenburg, Germany) utilizing Cu Kα1 radiation (1.5406 Å) and operating at 40 kV and 15 mA. XRD patterns were recorded between 10 and 80° 2θ at a step size of 0.01°. Rietveld refinement was performed using the Rigaku software. The specific surface area (SSA) was determined by the nitrogen adsorption–desorption isotherms (Brunauer-Emmett-Teller, BET method,) in liquid nitrogen at 77 K using a Tristar II Plus (Micromeritics, Norcross, GA, USA) instrument after degassing for at least 3 h at 110 °C. The morphology of the nanoparticles was observed using transmission electron microscopy (TEM) in a Tecnai BioTWIN (Fei, Hillsboro, Oregon, USA) instrument operated with an acceleration voltage of 120 kV and equipped with a 2k × 2k Veleta OSiS CCD camera. For the TEM imaging, the nanoparticles were suspended in ethanol in a water-cooled cup horn system (VCX750, cup horn Part no. 630-0431, Sonics Vibracell, Newport, CT, USA) (10 min, 100% amplitude) and one drop of the suspension was deposited onto a carbon coated copper grid (400 mesh carbon film, S160-4, Agar Scientific, Essex, UK). The grid was dried at ambient temperature overnight. Size distribution and surface charge of CaP and CaP-loaded nanoparticles were evaluated by dynamic light scattering (DLS) and ζ-potential analysis, respectively, in a Malvern Panalytical Zetasizer Ultra instrument (Malvern, UK) at 100 μg/mL particle concentration. Fourier transform infrared spectroscopy (FTIR) was performed on as-synthesized CaP nanoparticles and freeze-dried CaP conjugates in an Cary 630 FTIR spectrometer (Agilent, Kista, Sweden) in a wavenumber range of 400–4000 cm^−1^ with a 2 cm^−1^ resolution. Freeze-drying was performed in a Savant SpeedVac Plus SC210A lyophilizer (Thermo Scientific, Göteborg, Sweden) equipped with a refrigerated vapor trap (RVT4101, Thermo Scientific, Göteborg, Sweden). Absorbance measurements at 225 nm were performed using an Analytik Jena Specord 210 Plus ultraviolet and visible (UV-vis) spectrophotometer (Jena, Germany). The absorbance at 225 nm (where maximum absorbance of CaP nanoparticles was observed) is monitored over time in a partially covered cuvette in order to monitor only the top suspension layer according to the method presented in Spyrogianni et al. [49].

### 3.2. Preparation of Nanoparticles Suspension and Macromolecules Adsorption

Nanoparticle suspensions were prepared in phosphate buffer saline (PBS) at physiological pH 7.4 at an initial concentration of 1000 μg/mL. More precisely, 5 mg of the nanoparticles were dispersed in 5 mL of PBS pH 7.4 in a conical falcon polystyrene tube of 15 mL, followed by vortex-mixing for 30 s and ultrasonication for 20 min in a water-cooled cup horn system (VCX750, cup horn Part no. 630-0431, Sonics Vibracell, Newport, CT, USA). Ultrasonic energy was provided in pulses (10s on, 2s off) at 60% amplitude. As the nanoparticles are prone to sedimentation, the ultrasonication was paused every 5 min and additional vortex-mixing was implemented for 10 s in order to optimize particle dispersion.

In order to establish the experimental protocol for loading biologics on CaP nanoparticles, bovine serum albumin (BSA, ≥96%, Sigma-Aldrich, Stockholm, Sweden, MW: 66430.3 g/mol) and bradykinin acetate salt (≥98%, Sigma-Aldrich, Stockholm, Sweden, MW: 1060.2 g/mol) were selected as model protein and peptide, respectively. BSA was selected as model protein because of its stability and its wide application in evaluating sustained drug release systems [55,56], whereas bradykinin is rather small and can act as model for the loading of larger ones. Further on, as a case study LL-37 antimicrobial peptide (LLGDFFRKSKEKIGKEFKRIVQRIKDFLRNLVPRTES, >95%, Innovagen, Lund, Sweden, MW: 4493.3 g/mol) was loaded on CaP nanoparticles and the antibacterial efficiency of the LL-37-CaP nanoconjugates was evaluated.

Suspensions of protein and peptide-loaded CaP nanoparticles in PBS pH 7.4 (200 μL sample volume) were prepared by the addition of 100 μL of dispersed CaP nanoparticles in PBS pH 7.4 to an equal volume of protein/peptide solution of initial concentration ranging from 200 to 2000 μg/mL. The suspensions were placed on a roller mixer (Stuart SRT9D, Staffordshire, UK) for gentle mixing at 60 rpm at room temperature for 1, 2, 4, 6 and 24 h to investigate the effect of time on adsorption. The particles were separated via centrifugation at 10,000 rpm for 20 min (particle separation was confirmed by monitoring the luminescence of the CaP:Eu nanoparticles in the supernatant that was absent) and the supernatant containing the macromolecule content that has not been adsorbed was collected for quantification using a Pierce bicinchoninic acid (BCA) protein assay kit (ThermoFisher Scientific, Göteborg, Sweden) according to manufacturer’s instructions. Absorbance was measured at 562 nm using a microplate reader (SpectraMax Plus, Molecular Devices, San Jose, CA, USA) and the amount of macromolecules was calculated from a calibration curve. The amount of protein/peptide was calculated from the difference between the initial concentration and the concentration of the supernatant. Furthermore, the loaded particles were washed once with PBS and re-dispersed in PBS. The amount of macromolecule after the washing was also quantified in the supernatant after centrifugation (10,000 rpm, 20 min) and was found negligible (≤ 5%) indicating the stability of the conjugates. The suspensions of the loaded particles were stored at 4 °C and used for further experiments within a week. The loading capacity of the nanoparticles was expressed as (mass of macromolecule adsorbed onto nanoparticles in mg)/(total mass of nanoparticles in g). For each macromolecule concentration, at least three independent triplicates (from different particle suspensions and macromolecule solutions) were made and the final loading is presented as arithmetic mean ± standard deviation of the independent sets of replicate measurements.

### 3.3. Proteolysis and Growth Curves Analysis

#### 3.3.1. Degradation Assay

A total of 0.5 µg LL-37, either loaded on nanoparticles or not, was subjected to Proteinase K degradation assay. Input samples were taken without Proteinase K added, as a positive control and is considered to be “100%” when calculating protein amounts. LL-37 was incubated in 20 mM Tris-HCl, pH 8.0 with a total of 20 ng Proteinase K (Qiagen, Hilden, Germany) added to the reaction. Samples were incubated at 37 °C until given timepoints where the reaction was stopped by the addition of SDS loading buffer (at a final concentration of 50 mM Tris-HCl pH 6.8, 2% SDS, 10% glycerol, 1% β-mercaptoethanol, 12.5 mM EDTA and 0.02% Bromophenol blue) and samples were then immediately boiled for 10 min at 98 °C.

#### 3.3.2. SDS-PAGE

Samples were loaded onto a NuPAGE 4%–12% Bis-Tris gel (Invitrogen, Carlsbad, CA, USA) and run in MES-buffer (Invitrogen) at 40 mA/gel. Since LL-37 is a small peptide, samples were run only halfway through the gel. The gels where then washed in Mill-Q water 3 times, 10 min before immersion in Imperial Protein Stain (Thermo Scientific) for 1 h. After that Mill-Q water was used to destain the gels before image acquisition on GelDoc XRS+ (Bio-Rad, Hercules, CA, USA). Bands were quantified using Image Studio Lite v. 5.2 (LI-COR Biosciences, Lincoln, NE, USA).

#### 3.3.3. Growth Curves

The antimicrobial properties of LL-37-loaded CaP_S_ nanoparticles were assessed against the Gram-negative *E. coli* (HVM52 strain) and Gram-positive *S. pneumoniae* serotype 4 strain TIGR4 (T4, ATCC BAA-334). *E. coli* was incubated under shaking at 200 rpm overnight at 37 °C in 2 mL of Luria Bertani (LB) medium. Pneumococcal strains were grown at 37 °C on blood agar plates incubated overnight with 5% CO_2_. Colonies were inoculated into C+Y medium supplemented with 1% *v*/*v* of a mixture of heat inactivated horse serum and glucose to optical density at 600 nm (OD_600_) 0.1, grown until exponential phase (OD_600_ = 0.5), and again diluted to 0.05 with the aforementioned medium [52]. Bacterial growth was monitored in honeycomb multiwell plates using a Bioscreen C MBR instrument (Growth curves OY, Turku, Finland) in which the OD_600_ is recorded. Each sample was analyzed in triplicate. Imaging of bacterial cultures after 2 h incubation with the CaP_S_-LL-37 at 37 °C after staining with SYTO9 Green Fluorescent Nucleic Acid Stain (Invitrogen) was performed with a Delta Vision Elite microscope (Applied Precision, Uppsala, Sweden) under a 100× magnification objective. Z-stack projection images were acquired (excitation of the particles at 350 nm wavelength and emission at 570 nm wavelength and excitation of bacteria at 491 nm wavelength and emission at 516 nm wavelength). Images were acquired using SoftWoRx imaging program (Applied Precision, Uppsala, Sweden) and analyzed with ImageJ software.

## 4. Conclusions

We show here the engineering of CaP nanoparticles by FSP and evaluate their loading capacity as carriers of biological drugs (proteins and peptides). Different parameters affecting the loading capacity of CaP nanoparticles are investigated using BSA and bradykinin as model biomacromolecules (protein and peptide, respectively). More precisely, incubation time and macromolecule concentration are varied and that affects the amount of biological drug on the nanocarrier surface. High loading values are achieved due to the large surface-to-volume ratio of CaP nanoparticles and the fractal-like agglomerate structure of flame-made nanoparticles. The biological drug loading protocol is further implemented for the LL-37 antimicrobial peptide, a biological drug currently involved in various clinical trials. The amount of LL-37 that may be efficiently loaded on the flame-made CaP nanoparticles is the highest reported so far. Moreover, loading of LL-37 on CaP offers protection against enzymatic degradation, while the peptide functionality and antimicrobial activity against both Gram-positive and Gram-negative pathogens is retained upon loading. Therefore, the high drug loading of the developed nanocarriers here will facilitate the delivery of biologicals at diseased sites at high concentrations and therefore improve therapeutic efficiency.

## Figures and Tables

**Table 1:** Flame spray pyrolysis (FSP) processing conditions (O_2_ dispersion gas flow rate, precursor flow rate and concentration) and calculated precursor concentration used for the synthesis of the calcium phosphate (CaP) nanoparticles, along with their specific surface area (SSA) measured by N_2_ adsorption and the corresponded primary particle diameter (d_BET_).

Sample	O_2_ Dispersion Gas Flow Rate (L/min)	Precursor Flow Rate (mL/min)	Ca and P Concentration (M)	Precursor Concentration in Flame (mmol_Ca+P_/L_O2disp_)	SSA (m^2^/g)	d_BET_ (nm)
CaP_L_	3	8	0.2	0.533	73	26
CaP_S_	8	3	0.1	0.0375	246	8

**Table 2:** LL-37 loading values for various nanocarriers of different sizes reported in the literature. LL-37 loading values achieved in the present study for CaP nanocarriers outperform all LL-37 loading values found in the literature. Minimum Inhibitory Concentration (MIC) values of LL-37 and of the nanoparticle dose for the presented nanocarriers are also shown.

Nanocarrier	Hydrodynamic Diameter (nm)	Pathogen	LL-37 Loading (mg/g particle)	LL-37 MIC (μg/mL)	NPs MIC (μg/mL)	Ref
PLGA	163	*E. coli* (ATCC 25922)	1.02 ± 0.06	No significant antimicrobial activity		[37]
Nanostructured Lipid Carriers	220.6	*E. coli* (ATCC 25922)	16.76 ± 0.07	20 (72% killing)	1193	[36]
Solid Lipid nanoparticles	232.2 (loaded particles)	*E. coli* (ATCC 25922) *S. aureus* (ATCC 25923)	8.48–16.32	2–3 (65–72% killing)	184–236	[32]
				2–3 (42–47% killing)		
Au	10.47 ± 1.89	Methicillin-resistant *S. aureus*	0.56	0.9	1536	[45]
Mesoporous SiO_2_	307.9 * 294.6 *	*E. coli* (ATCC 25922)	24.1 ** 129.7 **	2.2	93	[31]
				44.9	346	
Calcium Phosphate	230 (d_BET_ = 8 nm)	*S. pneumoniae* (T4, ATCC BAA-34) *E. coli* (HVM52)	813.4 ± 101.3	50	61	This work
				200	246	

* as determined by SEM. ** Calculated assuming SiO_2_ particle density 2.2 g/cm^3^ [46]. Detailed calculations are presented in Supplementary Materials.

## Supplementary Materials

The following are available online. Table S1. Calculation of loading capacity from the literature, Table S2. LL-37 release at pH 7.4 after 2, 6 and 24 h at 25 °C and 37 °C, Figure S1. Effect of concentration of BSA and bradykinin on the loading capacity of CaP_L_ nanoparticles, Figure S2. Size distribution of CaP_S_ nanoparticles (intensity % data), Figure S3. Size distribution and *ζ*-potential of CaP_L_ nanoparticles, Figure S4. *ζ*-potential of BSA, bradykinin and LL-37 vs pH, Figure S5. Absorbance values of LL-37-loaded CaP_S_ nanoparticles, Figure S6. Absorbance values at 600 nm of LL-37-loaded CaP_S_ nanoparticles and of pure LL-37 in LB and C+Y media, Figure S7. Effect of CaP_S_ nanoparticle presence on bacterial growth.
