# Supplementary material for: Flame-Made Calcium Phosphate Nanoparticles with High Drug Loading for Delivery of Biologics
Source: Molecules. 2020 Apr 10;25(7):1747. doi: 10.3390/molecules25071747 (PMC7181047; doi:10.3390/molecules25071747)
Supplement: Supplementary file 1 [file molecules-25-01747-s001.pdf]

molecules

Supplementary Materials

Flame-Made Calcium Phosphate Nanoparticles with High Drug Loading for Delivery of Biologics

Vasiliki Tsikourkitoudi [1], Jens Karlsson [1], Padryk Merkl [1], Edmund Loh [1,3], Birgitta Henriques-Normark [1,2,3] and Georgios A. Sotiriou [1,*]

1 Department of Microbiology, Tumor and Cell Biology, Karolinska Institutet, SE-1 71 77 Stockholm, Sweden; vasiliki.tsikourkitoudi@ki.se (V.T.); jens.karlsson@ki.se (J.K.); padryk.merkl@ki.se (P.M.); edmund.loh@ki.se (E.L.); birgitta.henriques@ki.se (B.H.N.)

2 Department of Clinical Microbiology, Karolinska University Hospital, SE-171 76 Stockholm, Sweden

3 Lee Kong Chian School of Medicine (LKC) and Singapore Centre on Environmental Life Sciences Engineering (SCELSE), Nanyang Technological University, 639798, Singapore

* Correspondence: georgios.sotiriou@ki.se

Table S1. Calculation of loading capacity of mesoporous SiO_2 nanoparticles in mg LL-37/g particle according to data presented in Braun *et al.* [1].

Particles	Diameter (nm)	Radius (nm)	Particle Volume (m^3)	Mass of a single particle (g)	Number of Particles in 5 mg
**NSN**	307.9	153.95	$1.53 \cdot 10^{-20}$	$3.36 \cdot 10^{-14}$	$1.49 \cdot 10^{11}$
**MSNc**	294.6	147.3	$1.34 \cdot 10^{-20}$	$2.94 \cdot 10^{-14}$	$1.69 \cdot 10^{11}$

Particles	Adsorption (µmol LL-37/particle) [1]	µmol LL-37 in 5 mg	mg LL-37 in 5 mg (MW_{LL-37}=4493.3 g/mol**)	mg LL-37 / g particle
**NSN**	$1.8 \cdot 10^{-13}$	0.0268	0.120	24.06
**MSNc**	$8.5 \cdot 10^{-13}$	0.144	0.649	129.74

* Assuming density of SiO_2 2.2 g/cm³ [2].

**According to the provider.

Table S2. LL-37 release at pH 7.4 after 2 h, 6 h and 24 h at 25°C and 37°C. After 24 h, there is 1.1 % and 1.2 % of LL-37 released at 25°C and 37°C, respectively.

Temperature (°C)	Time (h)	% LL-37 released
25	2	0.3
	6	0.6
	24	1.1
37	2	0.5
	6	0.8
	24	1.2

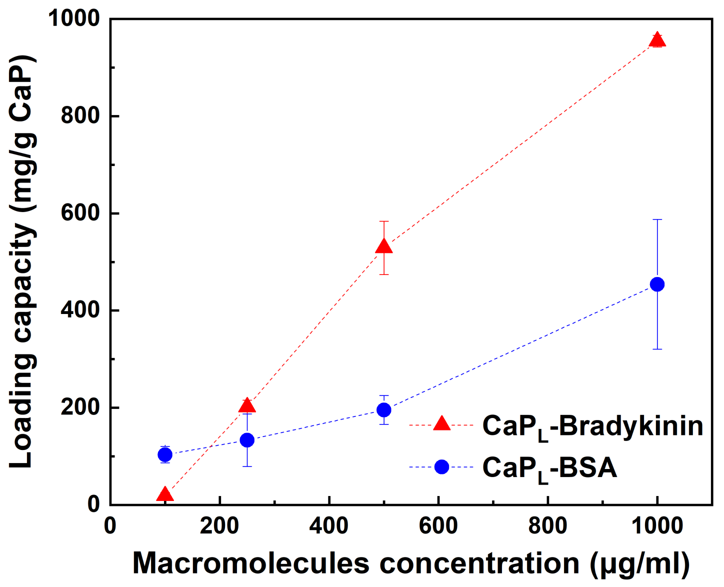

Figure S1. Effect of concentration of BSA and Bradykinin on the loading capacity of CaP_L nanoparticles after incubation for 6 h at room temperature (PBS pH 7.4, particle concentration 500 µg/ml). Data are reported as mean ± standard deviation, for at least 3 independent triplicates.

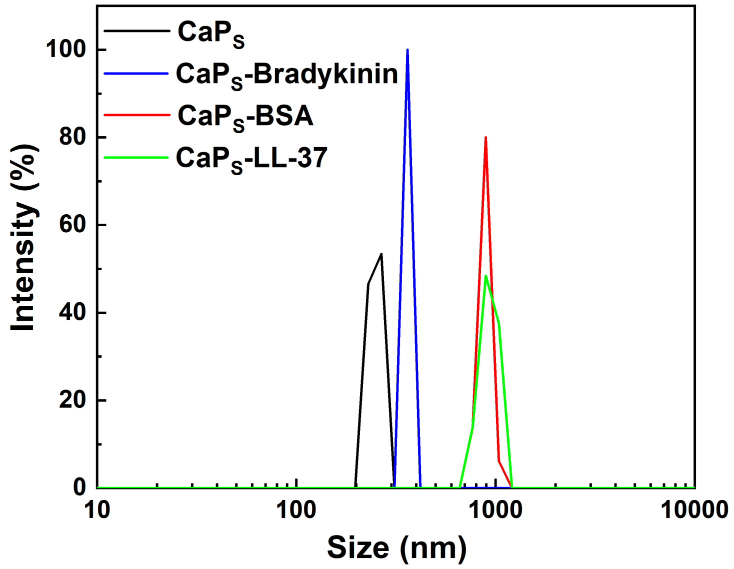

Figure S2. Size distribution of CaP_S nanoparticles (intensity % data) before and after loading with Bradykinin, BSA and LL-37 in PBS pH 7.4, as determined by DLS measurements (particle concentration 100 µg/ml).

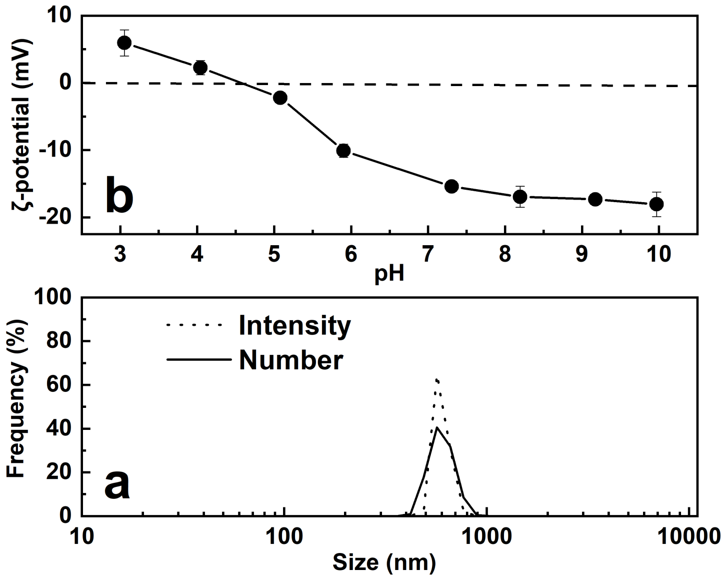

Figure S3. (**a**) Size distribution (both number and intensity data are presented) of CaP$_L$ nanoparticles in PBS pH 7.4 as determined by DLS measurements (particle concentration 100 µg/ml); and (**b**) ζ-potential profile of CaP$_L$ nanoparticles as determined by titration at different pH (particle concentration 100 µg/ml).

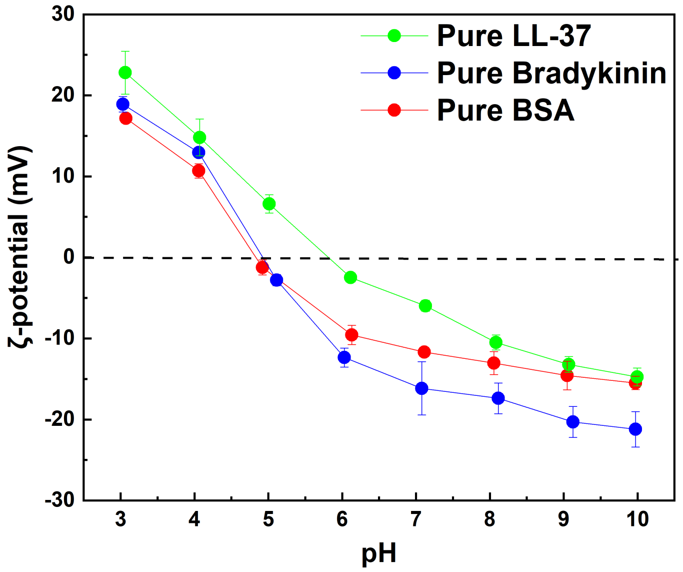

Figure S4. ζ-potential profile of pure BSA, bradykinin and LL-37 in PBS pH 7.4 as determined by titration at different pH (macromolecules concentration ~100 µg/ml). BSA and bradykinin have similar isoelectric points (~5), whereas the isoelectric point of LL-37 is ~6.

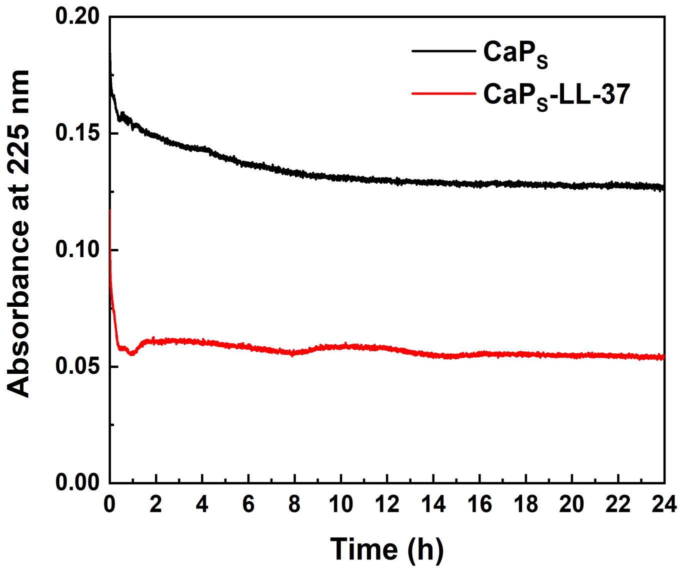

Figure S5. Absorbance at λ = 225 nm as a function of time for CaPs and CaPs-LL-37 nanocarriers of initial particle concentration 100 µg/ml in PBS. The light beam was aligned to monitor the absorbance of the top suspension layer. CaPs absorbance is stabilized after ~8 h whereas CaPs-LL-37 nanoparticles sedimented much rapidly (after ~2 h).

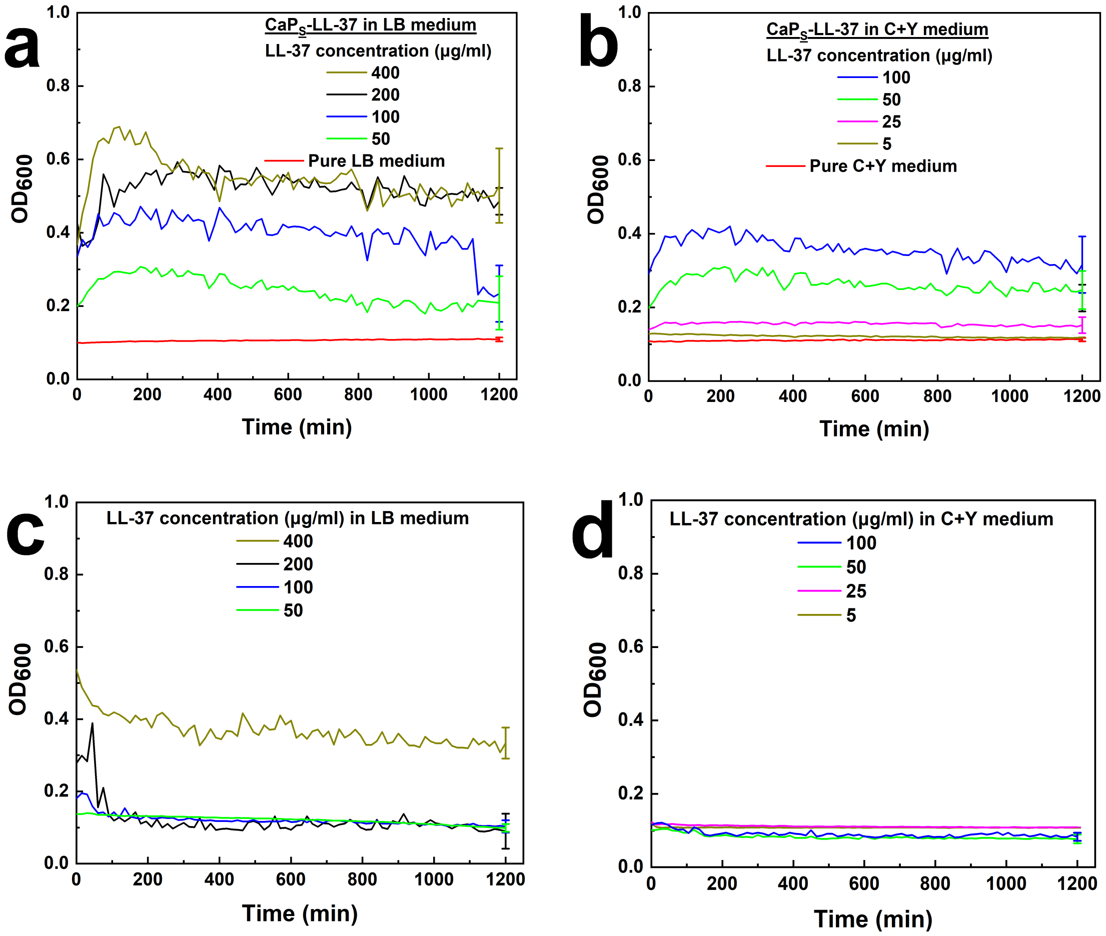

Figure S6. Absorbance values of LL-37-loaded CaPs nanoparticles in (**a**) LB medium and; (**b**) C+Y medium along with absorbance of pure media as measured in the bioscreen instrument at 600 nm; Absorbance values at 600 nm of pure LL-37 in LB (**c**) and C+Y (**d**) media. These values represent

background values that were used for the correction of the growth curves (Figure 7 of the main paper). Measurements had been performed in triplicate and mean values are presented with representative error bars.

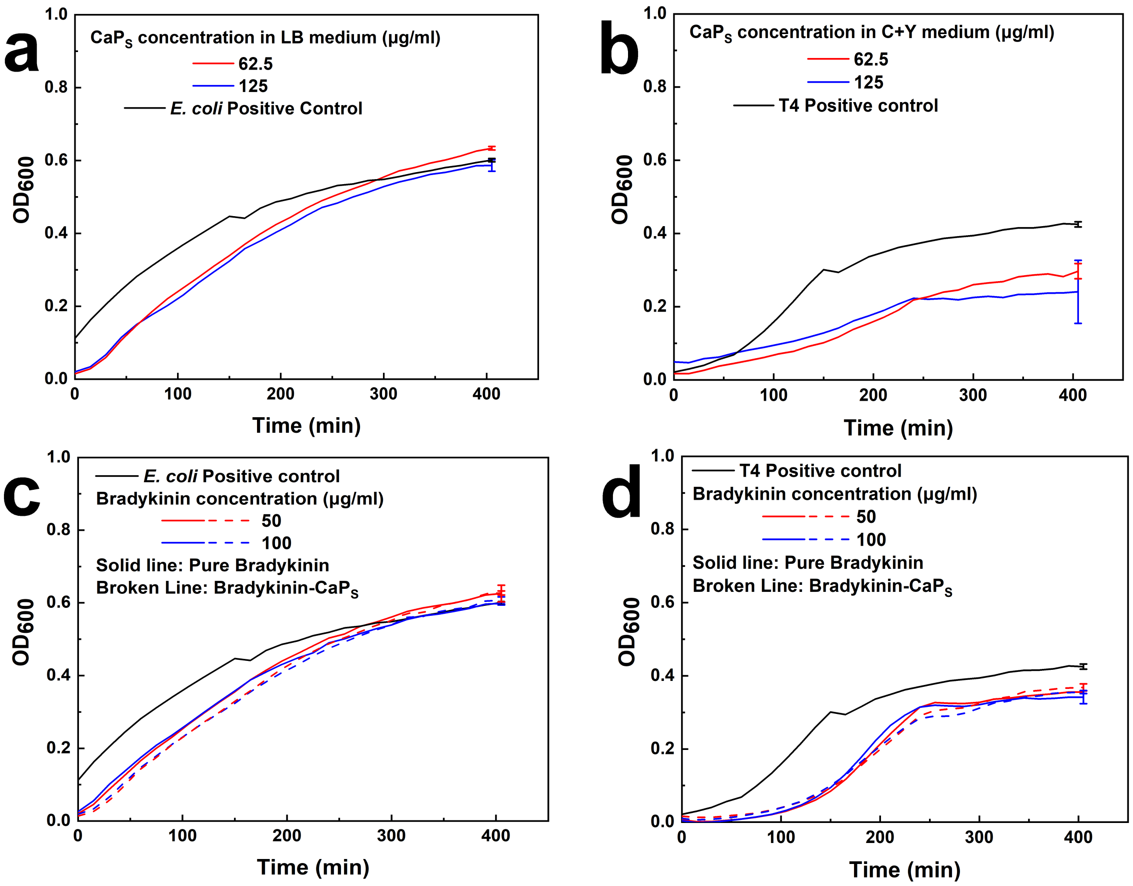

Figure S7. Effect of CaPs nanoparticle presence on (**a**) *E. coli* and (**b**) *S. pneumoniae* growth after subtraction of media absorbance (Figure S6a and b). Nanoparticle dose is calculated taken into consideration that LL-37 loading is ~800 mg/g particle. Thus, for 100 and 50 μg/ml LL-37 concentration, particle concentration is 125 and 62.5 μg/ml, respectively; *E. coli* (**c**) and *S. pneumoniae* (**d**) growth in the presence of pure bradykinin and bradykinin-loaded CaPs nanoparticles. Bradykinin was used as a control peptide in order to confirm that the observed antibacterial activity of the LL-37-loaded CaPs nanoparticles would be attributed to the presence of LL-37 and not to the nanoparticles. The observed antibacterial activity (Figure 7) is attributed to the LL-37 peptide and not to the CaPs nanoparticles. Measurements had been performed in triplicate and mean values are presented with representative error bars.

References

1. Braun, K.; Pochert, A.; Lindén, M.; Davoudi, M.; Schmidtchen, A.; Nordström, R.; Malmsten, M. Membrane interactions of mesoporous silica nanoparticles as carriers of antimicrobial peptides. *J. Colloid Interface Sci.* **2016**, *475*, 161–170.
2. Liu, J.; Zong, G.; He, L.; Zhang, Y.; Liu, C.; Wang, L. Effects of Fumed and Mesoporous Silica Nanoparticles on the Properties of Sylgard 184 Polydimethylsiloxane. *Micromachines* **2015**, *6*, 855–864.
